# Preliminary osteogenic and antibacterial investigations of wood derived antibiotic-loaded bone substitute for the treatment of infected bone defects

**DOI:** 10.3389/fbioe.2024.1412584

**Published:** 2024-07-09

**Authors:** Francesca Salamanna, Angela De Luca, Filippo Vandenbulcke, Berardo Di Matteo, Elizaveta Kon, Alberto Grassi, Alberto Ballardini, Giacomo Morozzi, Lavinia Raimondi, Daniele Bellavia, Viviana Costa, Stefano Zaffagnini, Milena Fini, Gianluca Giavaresi

**Affiliations:** ^1^ Surgical Science and Technologies, IRCCS Istituto Ortopedico Rizzoli, Bologna, Italy; ^2^ Department of Biomedical Sciences, Humanitas University, Milan, Italy; ^3^ IRCCS Humanitas Research Hospital, Milan, Italy; ^4^ Department of Traumatology, Orthopaedics and Disaster Surgery, Sechenov University, Moscow, Russia; ^5^ 2nd Orthopedic and Traumatologic Clinic, IRCCS Istituto Ortopedico Rizzoli, Bologna, Italy; ^6^ GreenBone Ortho SpA, Faenza, Italy; ^7^ Scientific Direction, IRCCS Istituto Ortopedico Rizzoli, Bologna, Italy

**Keywords:** biomaterials, device, antimicrobial activities, osteointegration, regenerative medicine

## Abstract

**Introduction:** The development of reliable treatments for infected or potentially infected bone loss resulting from open fractures and non-unions is extremely urgent, especially to reduce the prolonged courses of antimicrobial therapy to which affected patients are subjected. Numerous bone graft substitutes have been used over the years, but there are currently no effective solutions to treat critical bone loss, especially in the presence of infection. The present study evaluated the use of the biomorphic calcium phosphate bone scaffold b. Bone™, based on a next-generation resorbable biomimetic biomaterial, in bone reconstruction surgery in cases of infection.

**Methods:** Using an “*in vitro* 3D bone fracture model” to predict the behavior of this drug delivery system during critical bone loss at an infected (or potentially infected) site, the effects of scaffolds loaded with gentamicin or vancomycin on the viability and differentiation capacity of human mesenchymal stem cells (hMSCs) were evaluated.

**Results:** This scaffold, when loaded with gentamicin or vancomycin, exhibits a typical drug release curve that determines the inhibitory effects on the growth of *Staphylococcus aureus, Enterococcus faecalis*, and *Escherichia coli*, as well as relative biofilm formation.

**Discussion:** The study demonstrates that b.bone scaffolds can effectively address key challenges in orthopedic surgery and patient care by inhibiting bacterial growth and biofilm formation through rapid, potent antibiotic release, reducing the risk of treatment failure due to resistance, and providing a promising solution for bone infections and improved patient outcomes. Future studies could explore the combination of different antibiotics on these scaffolds for more tailored and effective treatments against post-traumatic osteomyelitis pathogens.

## 1 Introduction

Bone graft substitutes have been considered a major challenge by researchers from the launch of the first commercial CaP-based bone grafts 40 years ago to the present (Habraken et al., 2016; Wang and Yeung, 2017). Over the years, bone tissue engineering (BTE) has evolved in response to clinicians’ demand to find bone substitutes that increasingly resemble the physiological structure of bone to promote good osseointegration and subsequent bone healing (Roffi et al., 2017). Therefore, increasingly complex, and structured scaffolds have been created, as shown by the impressive literature on the subject (Wang and Yeung, 2017). In particular, the not-sintered hydroxyapatite and beta-tricalcium phosphate biomaterial derived from rattan wood is produced through a multi-step process aimed at converting the hierarchical structures of wood into biomimetic calcium phosphate scaffolds for bone tissue engineering (Tampieri et al., 2009). The b. Bone scaffold (GreenBone Ortho S. p.A.) is composed of biomimetic substituted calcium phosphate phases (HA 85%+ ß-TCP 15% and ions) (Tampieri et al., 2009). The good mechanical properties of the scaffold and its resistance to compressive and torsional forces depend on its composition, porosity, shape, and crystallinity (Ruffini et al., 2013; Filardo et al., 2014; Minardi et al., 2015). This innovative resorbable bone graft has proven to be a biomimetic scaffold whose geometry mirrors the physiological and anatomical bone structure, significantly improving bone regeneration and angiogenesis. A preclinical study on the safety and performance of the b. Bone implant for segmental bone reconstruction showed good integration with the host tissue with evidence of repair potential exceeding that of the allograft implant (Filardo et al., 2014).

Unfortunately, the opportunistic pathogen infections are one of the most common and dangerous complications associated with the use of implant devices. *Staphylococcus* is the one that most frequently involves an orthopedic infection (Pietrocola et al., 2022) resulting in posttraumatic osteomyelitis for almost 80% of all cases of human disease (Zelmer et al., 2022). *Pseudomonas, Enterococcus, Streptococcus* and the *Enterobacteriaceae* family have also been isolated from patients with such implants (Schulze et al., 2021). These data place bone and joint infections (BJI) as a significant cause of morbidity and occasional mortality, as well as increased costs for patient treatment, thus remaining an open challenge (Matar et al., 2021). Despite the continuous improvement in surgical techniques and antibiotic prophylaxis, bacterial infections have a high incidence (Nelson et al., 2023). In addition, the colonization of implant surfaces and the formation of a bacterial biofilm protect the microorganisms and confer resistance to both natural defenses of the organism and antibiotic therapy (Dastgheyb et al., 2015). It is therefore crucial to adopt resorbable materials loaded with antibiotic agents exerting antibacterial activity and preventing the formation of bacterial biofilms on their surfaces (McKee et al., 2010; Sabater-Martos et al., 2023; Steadman et al., 2023).

The results obtained to date with the use of b. Bone scaffold on bone regeneration make this material suitable for the treatment of infections. For this study, a modified b. Bone scaffold was loaded with the antibiotics vancomycin and gentamicin, in order to evaluate in an *in vitro* 3D bone fracture model the impact of local release of antibiotics on bone regeneration, bacterial eradication and biofilm inhibition.

## 2 Materials and methods

### 2.1 Fabrication of modified b.Bone scaffold

**FIGURE 1:**
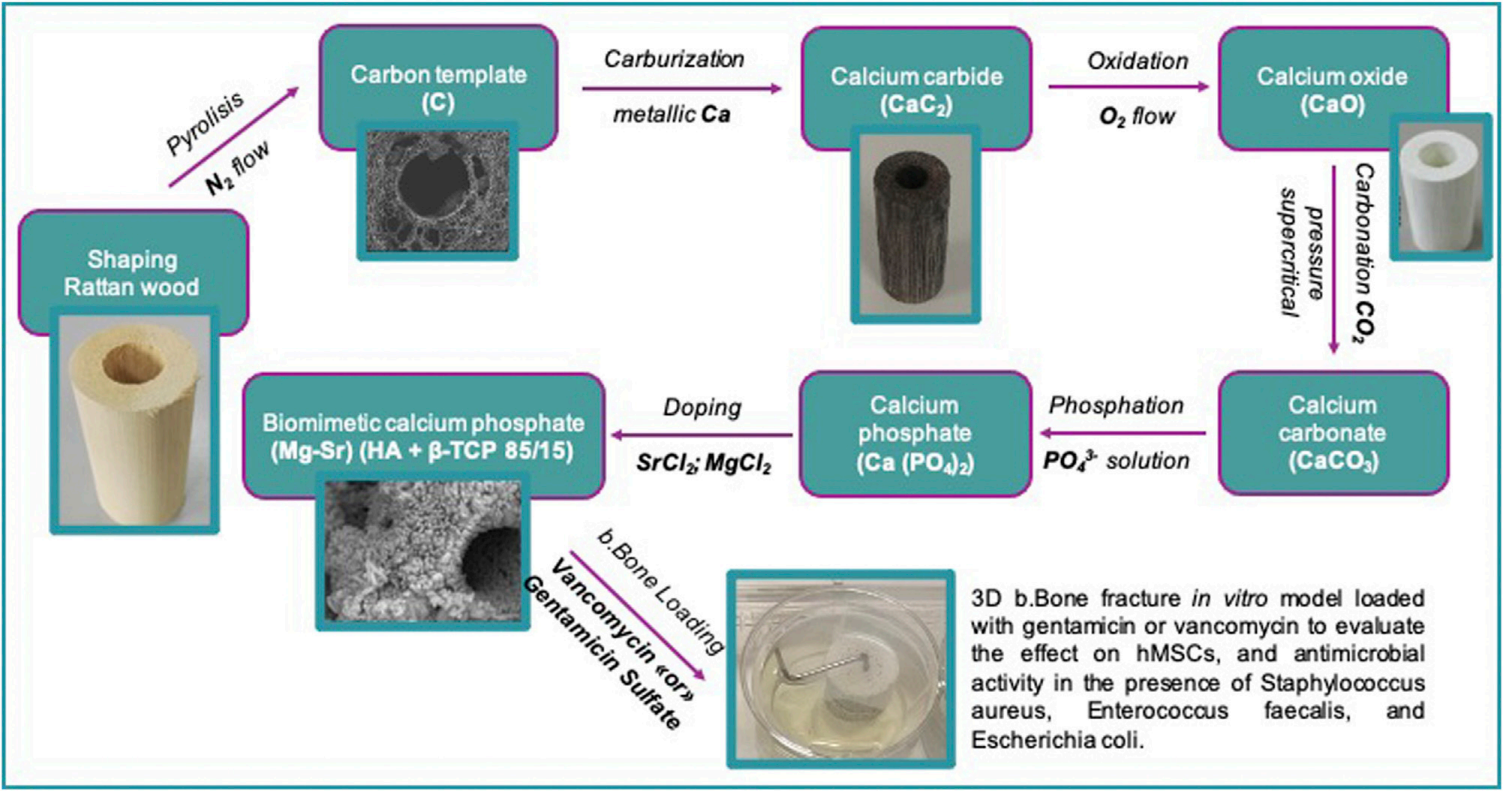
The b.Bone manufacturing process and loading flow chart.

Rattan wood pieces (Calamus manan) were used as 3-D templates and shaped into suitable dimensions to obtain a final cylinder of diameter of 10 mm. The wood was heated at 900°C (heating/cooling rate: 1°C min^−1^) under nitrogen flow, to eliminate the organic components and obtain a carbon template. The obtained carbon template was then placed in a vessel containing metallic Ca granules (Sigma Aldrich, United States), and subjected to heating up to 1200°C (heating/cooling rate: 2°C min^−1^) in a vacuum furnace to allow sublimation of Ca in an argon atmosphere (*p* = 100 mbar). The resulting CaC_2_ template was converted into CaO by heating at 1100°C under air flow. Then, further conversion of the resulting CaO into CaCO_3_ was carried out by heating under a pressure of carbon dioxide under non-isothermal and isobaric conditions (*p* = 100 atm) from room temperature to 800°C. This process was carried out under wet conditions, derived from hydrated carbon dioxide with a molar ratio of CO_2_/H_2_O from 8 to 10. During the process, CO_2_ is maintained in the supercritical state, activating the reaction mechanism. Then, the obtained CaCO_3_ structure was placed in a closed reactor containing a 3.0 M solution of (NH_4_)_2_HPO_4_ (Sigma Aldrich, United States) buffered at pH 8.5, and heated at 220°C under water vapour pressure (∼20 bar) to activate a dissolution-reprecipitation reaction, converting CaCO_3_ into calcium phosphate. Finally, a conclusive treatment by soaking in an aqueous solution containing 0.5 M SrCl_2_ and 0.5 M MgCl_2_ ions for 24 h at 50°C, buffered at pH 7.0, was carried out to obtain a biphasic (hydroxyapatite (HA) and β-tricalcium phosphate (β-TCP)) biomorphic apatite scaffold containing doping with Mg^2+^, Sr^2+^ and CO_3_^2-^ ions. Finally, the cylinders of 10 mm diameters were cut into disks of 3 mm and 4 mm height and sterilized with gamma radiation for the *in vitro* tests. With the same processes, were produced hollow cylinders with an external diameter of 25 mm, internal diameter of 13 mm and height of 10 mm for the dissolution tests. The manufacturing process is illustrated by a flowchart in Figure 1 and represents the same method process used to produce b. Bone (Sprio et al., 2021).

#### 2.1.1. Material characterization

Biphasic calcium phosphate granules were coated with gold using a Desk V Sputter (Denton Vacuum), and the surface topographies were examined by secondary electrons using a SEM (Thermo Fisher Scientific Phenom XLG2) at an acceleration voltage of 15 keV and 30 mA.

Porosity of the material was calculated through the formula:

$$P=100-\left(d_{c}/d_{th}*100\right)$$

Where P is the porosity percentage, d_c_ is the calculated density of the material through the formula mass/volume and d_th_ is the theorical density of the material, calculated through the formula:

$$d_{th}=\frac{\frac{{MF}_{HA}}{d_{HA}}}{\frac{{MF}_{HA}}{d_{HA}}+\frac{{MF}_{\beta TCP}}{d_{\beta TCP}}}*d_{HA}+\frac{\frac{{MF}_{\beta TCP}}{d_{\beta TCP}}}{\frac{{MF}_{HA}}{d_{HA}}+\frac{{MF}_{\beta TCP}}{d_{\beta TCP}}}*d_{\beta TCP}$$

where MF_HA_ is the mass fraction of HA, MF_βTCP_ is the mass fraction of β-TCP, d_HA_ is the theorical density of HA (3.15 g/cm^3^) and d_βTCP._

Physico-chemical characterization was carried out on samples crushed into fine powders by mortar and pestle. The phase composition was obtained by X-ray diffraction (XRD), using a Aeris diffractometer (Malvern Panalytical; CuKα radiation: λ = 1.54178 Å). XRD spectra were recorded in the 2θ range of 10°–80° with a counting time of 0.5 s and a step size of 0.02°. XRD spectra were subjected to full profile analysis (HighScore Plus) to determine phase composition.

Chemical analysis was performed on dried samples using an ICP-OES spectrometer (Agilent 5100). About 20 mg of the sampling material was dissolved in 2 mL of nitric acid (Sigma Aldrich; 65 vol%) and then diluted with double-distilled water to obtain 100 mL of solution. The solution was then analysed using a standard prepared from primary standards (1000 ppm, Sigma Aldrich) to determine Mg, Ca, P and Sr content. The Ca and P content was elaborated as Ca/P molar ratio while Mg and was expressed as weight percentage.

### 2.2 Antibiotic-loaded protocols and release

Scaffolds were used plain or loaded with gentamicin sulfate at a concentration of 5 mg × 0.4 g disc weight (Bio Basic Inc.; Toronto, Canada) or with vancomycin hydrochloride 10 mg × 0.4 g disc weight (Hikma Italia S.p.A., Pavia, IT). Briefly, the solutions of gentamicin sulfate and vancomycin hydrochloride were reconstituted to a final concentration of 50 mg/mL and 100 mg/mL respectively, and 100 μL of each solution were dispensed separately onto different discs. Both solutions were completely absorbed by the porosity of the scaffolds.

Antibiotic release was assessed over 3 weeks, in particular vancomycin and gentamicin release profiles were obtained by preparing 1 mg/mL of each antibiotic solution and dropping 1 mL of each onto the scaffold. After loading, the samples were stirred slowly at room temperature for 15 min. Three scaffolds loaded with vancomycin and 3 scaffolds loaded with gentamicin were then placed separately in 100 mL cylindrical containers containing 19 mL of phosphate buffered saline (PBS) and kept at 37°C with stirring. The vancomycin and gentamicin releases were analysed at the following time points: 30 min, 1 h, 2 h, 4 h, 6 h, 24 h, 48 h, 168 h, and 336 h. At each sampling time, 2 mL of solution was removed and replaced with 2 mL of fresh PBS solution to avoid saturation of vancomycin and gentamicin. Each solution was analyzed using a UV/VIS spectrophotometer (Perkin Elme, Lambda 750, United States) by measuring the absorbance at 280 nm and 207 nm, respectively.

The cumulative percentage release (%) at each time point was calculated according to the following formula:

$$\text{Cumulative percentage release }\%=\frac{C*V+\sum_{i}C_{i}V_{i}}{C\;\text{total}}*100$$

where C and *C*_*i*_ are respectively the concentration of the antibiotics in the mother medium at testing time point (t) and the concentration of the antibiotics in the sample drawn at testing time point (i); V is the volume of the mother medium at time t (20 mL) and *V*_*i*_ (2 mL at each tested point) is the volume of medium withdrawn at time point (i), and C total is the weight of each antbiotic (1 mg) loaded on scaffold at the volume (1 mL). The above experiments were repeated three times.

### 2.3 *In vitro* 3D bone fracture model

Human mesenchymal stem cells (hMSC, PoieticsTM Stem Cells, Lonza Walkersville, United States, batch 21TL116647, passage 2) derived from bone marrow were cultured in Mesenchymal Stem Cell Basal Medium (MSCBM, PoieticsTM Lonza Walkersville, United States) supplemented with 10% fetal bovine serum (FBS, Lonza, Verviers, Belgium), 100 U/mL penicillin, 100 μg/mL streptomycin (Gibco, Life Technologies, Carlsbad, CA) and 5 μg/mL plasmocin (Invitrogen, San Diego, CA), and incubated at 37 °C in a humidified 95% air/5% CO_2_ atmosphere (standard condition). Medium was changed every 3 e 4 days until 80%–90% confluence.

To generate the 3D bone fracture model 1 × 10^6^ hMSCs were seeded on plain scaffolds and on scaffolds loaded with 10 mg of vancomycin hydrochloride or with 5 mg of gentamicin sulfate. Subsequently, all scaffolds seeded with hMSCs were cultured with medium differentiating towards the osteogenic phenotype at 37.0°C ± 0.5°C with 95% of humidity and 5.0% ± 0.2% CO_2_ for 2 weeks. Osteogenic medium consisted of MSCBM supplemented with dexamethasone 10^–8^ M, ascorbic acid 50 μg/mL, and β-glycerophosphate 10 mM (Poietics™ Lonza Walkersville, United States). After 2 weeks, a plain scaffold, a scaffold loaded with 10 mg of vancomycin hydrochloride and a scaffold loaded with 5 mg of gentamicin sulfate (all without hMSCs) were sandwiched between two scaffolds of the same type previously seeded with hMSCs, simulating a 3D *in vitro* fracture model. This triple layer structure was held together by a stainless-steel wire inserted in the center and kept under standard culture conditions at 37°C ± 0.5°C with 95% humidity and 5% ± 0.2% CO_2_ for 2 additional weeks with osteogenic differentiation medium (Poietics™). At the end of the total 4 weeks, the 3D models were assessed for the analyses of cell viability, colonization, osteogenic differentiation, and pro-inflammatory markers expression.

#### 2.3.1 Cell viability

It was quantified at 7 and 14 days. Alamar blue dye (Serotec, Oxford, UK) was added to the 3D bone fracture model (1:10 v/v) and incubated for 4 h at 37°C. This non-toxic reagent allows evaluation of the cell activity at different endpoints by the chemical reduction of its main component resazurin into resorufin in the mitochondria of living cells. The fluorescent product was quantified at 530ex–590em nm wavelengths using a microplate reader (VICTOR X2030, Perkin Elmer, Milano, Italy) and expressed as relative fluorescence units (RFU).

#### 2.3.2 Cell colonization

Live/Dead^®^ assay (Molecular Probes, Eugene, OR, United States) was carried out according to the manufacturer’s instructions. Briefly, the scaffold sandwiched between the two scaffolds seeded with MSCs was rinsed in PBS and incubated with 2 μM Calcein AM and 4 μM EthD-1 for 45 min in the dark at room temperature. Then, samples were visualized using an inverted microscope equipped with an epifluorescence setup excitation/emission setting of 488/530 nm to detect green fluorescence (live cells) and 530/580 nm to detect red fluorescence (dead cells); images were captured at 4 and 10 × magnification.

#### 2.3.3 Osteogenic differentiation

Total RNA was extracted from hMSCs on the 3D models at 7 and 14 days of culture using Trizol^®^ reagent (AMBION by Life Technologies, Carlsbad, CA, United States) and chloroform (Sigma Aldrich) until harvesting the aqueous phase. The procedure was continued by using the commercial PureLinkTM RNeasy Mini Kit (AMBION), quantified by NANODROP spectrophotometer (NANODROP 2720, Thermal Cycler, Applied Biosystem) and reverse transcribed with SuperScriptVILO cDNA Synthesis Kit (Life Technologies), following the manufacturer’s instructions. The obtained cDNA was diluted to the final concentration of 5 ng/μL for each sample, considering the starting amount of RNA, to exploit the same range of amplification efficiency. Semi-quantitative polymerase chain reaction (PCR) analysis was performed for each sample, in duplicate, in LightCycler 2.0 Instrument (Roche Diagnostics GmbH, Manheim, Germany) using QuantiTect SYBR Green PCR Kit (Qiagen, Hilden, Germany) and gene-specific primers (Supplementary Table S1). The protocol included a denaturation cycle at 95°C for 15′, 25 to 40 cycles of amplification (95°C for 15″, 55°C annealing temperature for each target for 20″, and 72°C for 20″). After melting curve analysis to check for amplicon specificity, the threshold cycle was determined for each sample and relative gene expression was calculated using the 2^−ΔΔCt^ method, with GAPDH as reference gene and unloaded scaffold at each endpoint as calibrators.

#### 2.3.4 Immunoenzymatic assays

Proinflammatory cytokines produced by cells within the 3D model were measured by ELISA colorimetric tests for the quantification of Interleukin 6 (IL6, SEA079Hu Cloud-Clone Corp, United States), Interleukin 1β (IL1β, EK0392, Boster Bio, Pleasanton, CA, United States), and Tumor Necrosis Factor α (TNFα, SEA133Hu, Cloud-Clone Corp., Katy, TX, United States). Supernatants were collected from all wells at 7 and 14 days and maintained at −20°C until used.

### 2.4 Antibacterial activities

Scaffold antibacterial activity was measured against different Gram-positive bacterial strains -*Staphylococcus aureus* (ATCC 25923; 33592; BAA2856), and *Enterococcus faecalis* (ATCC 29212; 49532) – and Gram-negative bacterial strain of *Escherichia coli* (ATCC 25922). Briefly, each bacterial strain was pre-cultivated in Luria Bertani medium (LB – composed of 10 g sodium chloride, 10 g tryptone, and 5 g yeast extract per liter) and incubated overnight at 37°C with shacking (180 rpm). Afterward, each strain was adjusted at several concentrations using the measurement of optical density at 600 nm.

#### 2.4.1 Bacterial growth inhibition test

Antimicrobial tests were performed on unloaded and loaded discs with two different antibiotics (gentamicin or vancomycin). For each bacterial strain *Staphylococcus aureus* (ATCC 25923 and ATCC 33592), *E. faecalis* (ATCC 29212), and *E. coli* (ATCC 25922), the unloaded (n = 3) and loaded antibiotic (n = 3 for each antibiotic) scaffolds were transferred to different 15 mL steril tubes and the bacterial suspension was added to the final concentration of 1 × 10^8^ colony-forming units per milliliter of culture (CFU mL^−1^). Untreated bacteria grown in LB medium were considered as a growth control. All samples were incubated at 37°C for 24 h under shacking conditions (180 rpm) before plating onto LB agar plates for colony counting. Three replicates were used for each condition. Turbidity was measured by spectrophotometric analysis at an optical density of 600 nm (OD) read by using a UV-visible spectrophotometer Jasco 7850 (Lecco, Italy, JascoEurope), indicating the presence and rate of bacterial overgrowth relative to controls.

To obtain the total number of each bacterial strain grown in the culture broth and adhering to the device surface, the culture broth was quantified after that the unloaded and loaded discs were vortexed for 5 min and sonicated for 10 min at 30 Hz to favor the bacteria detachment from the surface. Then, 10 µL of serial dilutions of the solution sonicated were spotted onto LB agar plates. Plates were incubated at 37°C overnight. Subsequently, the colony forming units (CFU) were determined, indicating the number of viable bacteria present in the suspension (liquid broth and bacteria adhering to the surface of the device). Three replicates will be considered for each measurement. All the reagents were purchased from Sigma-Aldrich (Milan, Italy).

#### 2.4.2 Zone of inhibition test (ZOI)

The ability of the tested antibiotics to diffuse out of the scaffold and prevent bacterial growth was assessed by ZOI test or “agar diffusion test”. *Staphyloccocus aureus* (ATCC 25923 and ATCC 33592), *E. faecalis* (ATCC 29212), and *E. coli* (ATCC 25922) bacterial strains (final concentration ∼1 × 10^9^ CFU/mL) were plated onto Muller Hinton (MH) agar plates (Thermo Scientific Italia, c/o Segreen Business Park, Via San Bovio 3, 20054 Segrate – MI, Italy) to obtain a confluent growth. For each bacterial strain, unloaded (n = 3) and loaded with antibiotic (n = 3 for each antibiotic) discs were placed on MH plates and incubated at 37°C overnight. The diameter of the bacterial inhibition zone was measured with a caliper for each disc placed on the MH plates. Three replicates will be considered for each measurement.

#### 2.4.3 Bacterial biofilm formation assay

To evaluate the biofilm formation, high biofilm former strains and not, were used, such as *Staphylococcus aureus* (ATCC 25923 and ATCC BAA 2856), *E. faecalis* (ATCC 49532) and *E. coli* (ATCC 25922). Strains were inoculated in LB medium (1 × 10^8^ CFU/mL) and 1 mL of bacterial suspension was added onto plain (n = 4) or antibiotic-loaded (n = 4 for each antibiotic) discs for each selected time. The samples were incubated at 37°C for 15, 30, 60 and 120 min and overnight; at each time point, non-adherent bacteria were removed by washing with PBS, while adherent bacteria were mechanically removed by sonication and plated for colony forming unit (CFU) counts. Bacterial biofilm formation and bacterial viability were evaluated by scanning electron microscope (SEM) analysis. After 24 h in culture, the discs were washed with PBS to remove the excess of bacteria not adhered to the disc and residual broth medium. The samples were fixed in 2.5% glutaraldehyde (v/v) in pH 7.4 phosphate buffer 0.1 M for 3 h at room temperature and dehydrated through three washing steps (10 min each) with increasing concentrations of ethanol (30, 50, 70, 95% v/v) and absolute ethanol for 1 h. After having completed dehydration, the specimens were dried at room temperature and were gold-coated (B7340 Manual Sputter Coater Assing S. p.A) to make them conductive. SEM images were obtained at 20 kV using backscattered electron (BSE) imaging by EVO HD 15 Zeiss SEM (Carl Zeiss Microscopy, GmbH 07745 Jena, Germany).

### 2.5 Statistical analyses

Statistical analysis was performed with GraphPad Prism software 9.5.1. Data are reported as Mean ± standard deviations (SD) at a significance level of *p <* 0.05. After checking for normal distribution and homogeneity of variance, one-way ANOVA or two-way ANOVA was used to compare the microbiological results between the antibiotic-loaded scaffolds (plain, gentamicin-loaded and vancomycin-loaded) or to compare cell viability and gene expression between the antibiotic-loaded scaffolds (plain, vancomycin-loaded and gentamicin-loaded) over the experimental times. Finally, Sidak’s *post hoc* multiple comparison test was performed to detect significant differences among groups.

## 3 Results

### 3.1 Material characterization

**FIGURE 2:**
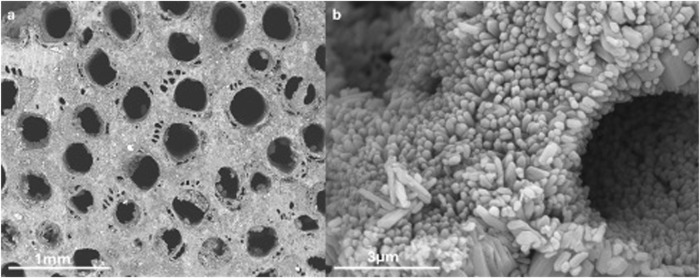
SEM image of the material showing the macropores **(A)** and the crystal dimensions **(B)**.

The SEM evaluation confirms the presence of the typical channels of Rattan architecture, both macroscopic and microscopic ones in accordance with the porosimetric distribution present in Tampieri et al., 2018; Tampieri et al., 2018). The dimensions of the crystals are in the range between 100 and 1000 nm (Figure 2).

**FIGURE 3:**
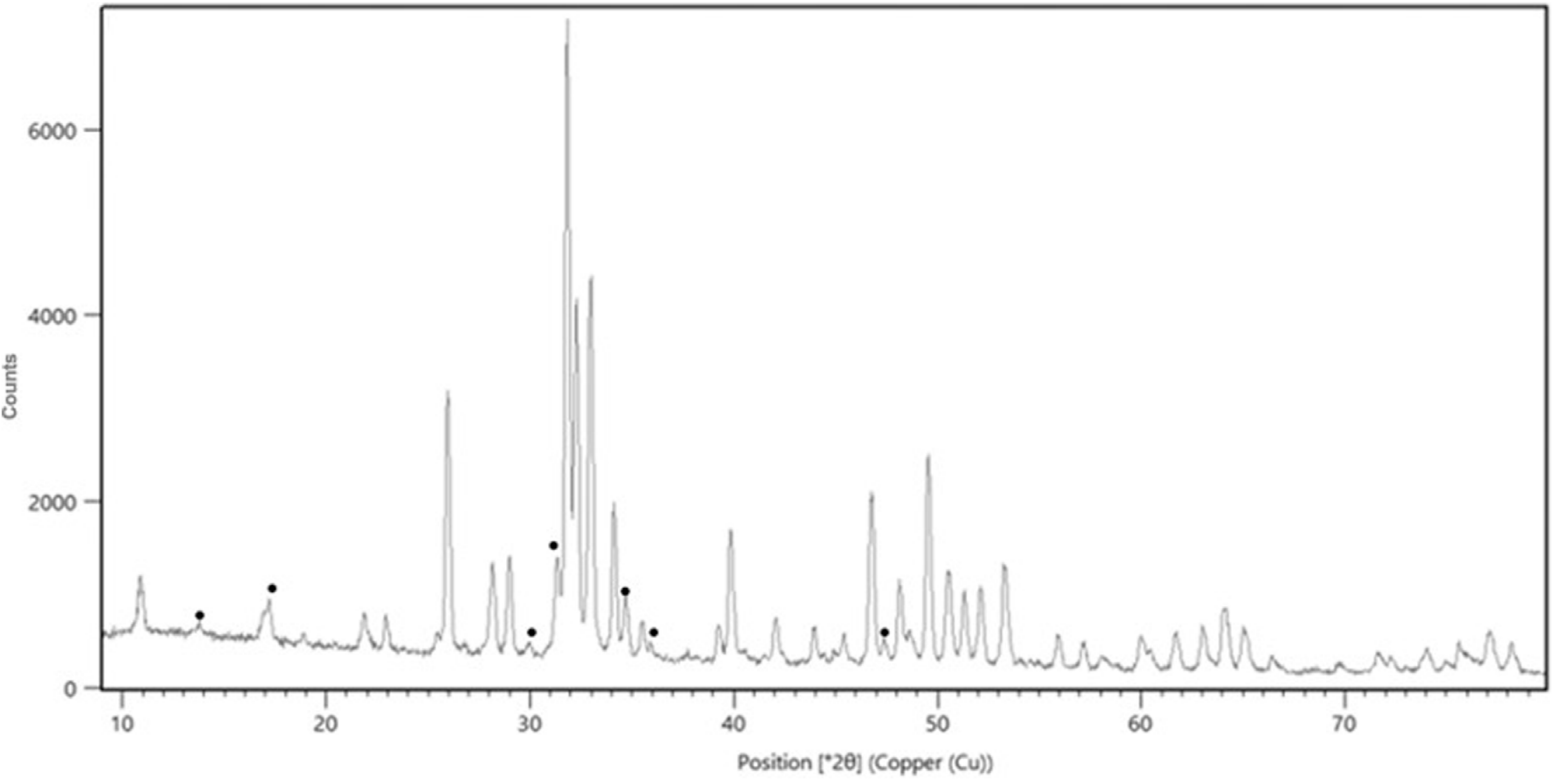
XRD pattern of the scaffold showing the biphasic composition. In the figure the peaks attributed to β-TCP have been marked with the symbol ●.

The porosity of the examined materials is 55% ± 2%. From Rietveld analysis of the XRD spectra was determined a biphasic composition of HA and β-TCP, without the presence of other phases (Figure 3; Supplementary Table S2). The weight percentage composition of the material is 85/15 ± 1. The chemical analysis shows a Ca/P ratio of 1.55 ± 0.03, a Mg weight % content of 0.57 ± 0.05 and a Sr weight % content of 0.73 ± 0.08.

### 3.2 Drug release

**FIGURE 4:**
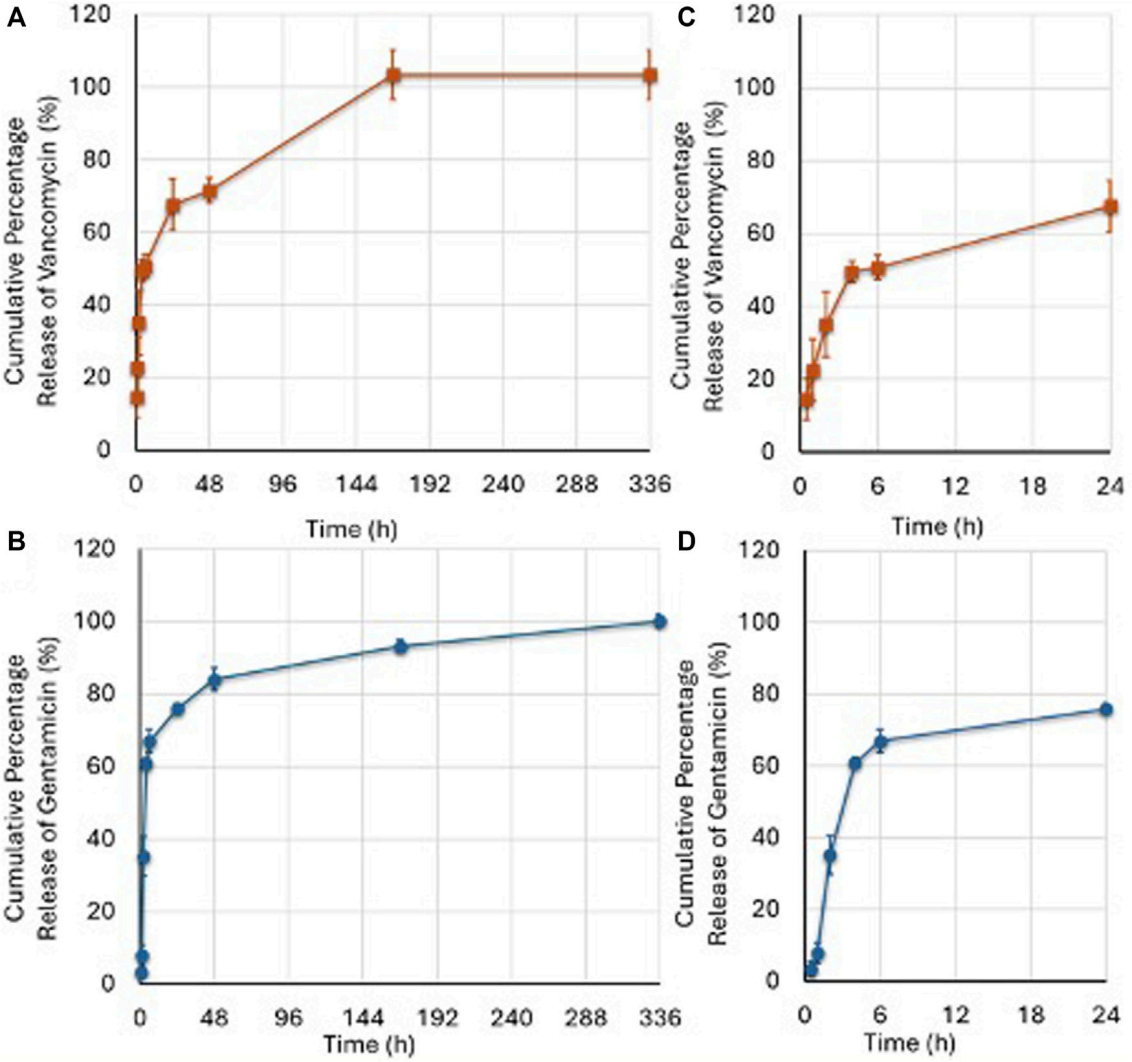
Vancomycin **(A)** and Gentamicin **(B)** released at different timepoints as percentage of the drug initially loaded. The amount of released vancomycin and gentamicin was calculated according to the amount (1 mg) loaded into the G. The **(C,D)** images represent the boost release of the two antibiotics over a 24-h period. (Mean ± SD, n = 3). Two-ANOVA (*F* = 134.9, *p* < 0.0005): Vancomycin release was higher than Gentamicin at 0.5 (*p* = 0.008); 1 (*p* = 0.008); and 168 (*p* = 0.014) hours; Gentamicin release was higher than Vancomycin at 4 (*p* = 0.008); 6 (*p* < 0.0005); 24 (*p* = 0.045); and 48 (*p* = 0.034) hours.

Analysis of the release of vancomycin and gentamicin showed a bimodal profile, as reported in Figure 4. An initial rapid release was observed in the first 6 h, leading to approximately 50% release of loaded vancomycin and 67% release of gentamicin, reaching levels of 67% and 76%, respectively, at 24 h, values shown in Supplementary Table S3. A second slower release leading to the complete release of the antibiotic in 168 h was registered. This bimodal release may be due to the porosity of the scaffold, which ranges from nanopores up to pores of some hundreds of micrometers. At the subsequent timepoint of 336 h, no additional amount of vancomycin or gentamicin was released.

### 3.3 *In vitro* 3D bone fracture model

**FIGURE 5:**
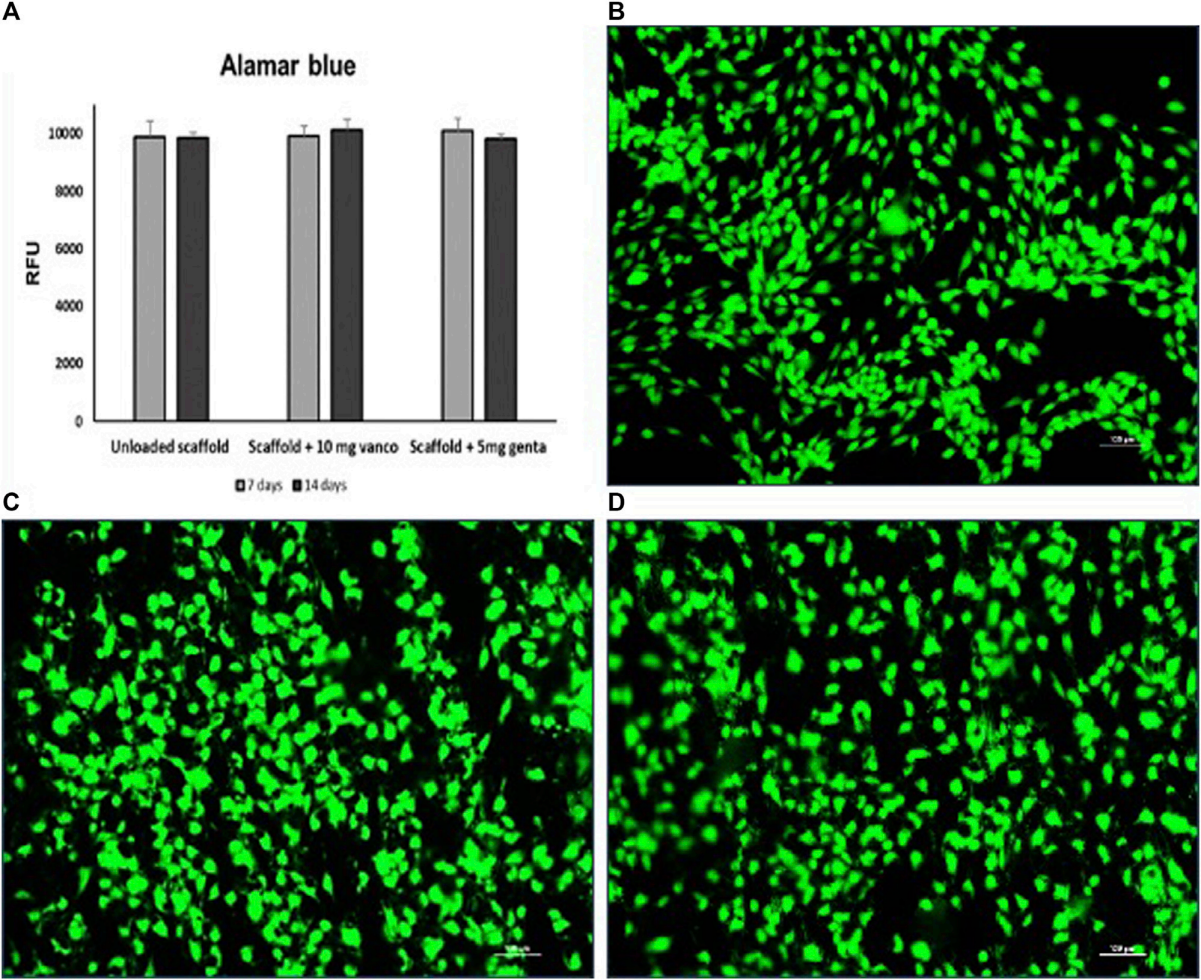
**(A)** Cell viability determined by Alamar blue staining of hMSCs grown on 3D model with scaffold loaded with 10 mg of vancomycin hydrochloride or 5 mg of gentamicin sulphate or unloaded scaffold. **(B–D)** Live and dead cell staining conducted on the scaffolds inserted between the two scaffolds previously seeded with hMSCs; **(B)** scaffold loaded with 10 mg of vancomycin hydrochloride, **(C)** scaffold loaded with 5 mg of gentamicin sulfate and **(D)** unloaded scaffold after 14 days of culture. Viable cells stain green while dead cells stain red: images show that cells grew regularly into the scaffolds as evidenced by the green-fluorescent dye in the absence of the red fluorescence dye (10 × magnification: bar = 100 μm).

Alamar blue test demonstrated the maintenance of cell viability at 7 and 14 days for all types of 3D models, not detecting any significant difference among the models (Figure 5A). Live/Dead^®^ assay demonstrated that after 14 days of culture of the 3D model the scaffolds inserted between the two scaffolds previously seeded with hMSCs and loaded with 10 mg of vancomycin hydrochloride or 5 mg of gentamicin sulfate or unloaded were colonized by hMSCs (Figures 5B–D).

The merged images showed that hMSC were viable and proliferated regularly in all porous scaffolds and inside the scaffold’s porosities.

**FIGURE 6:**
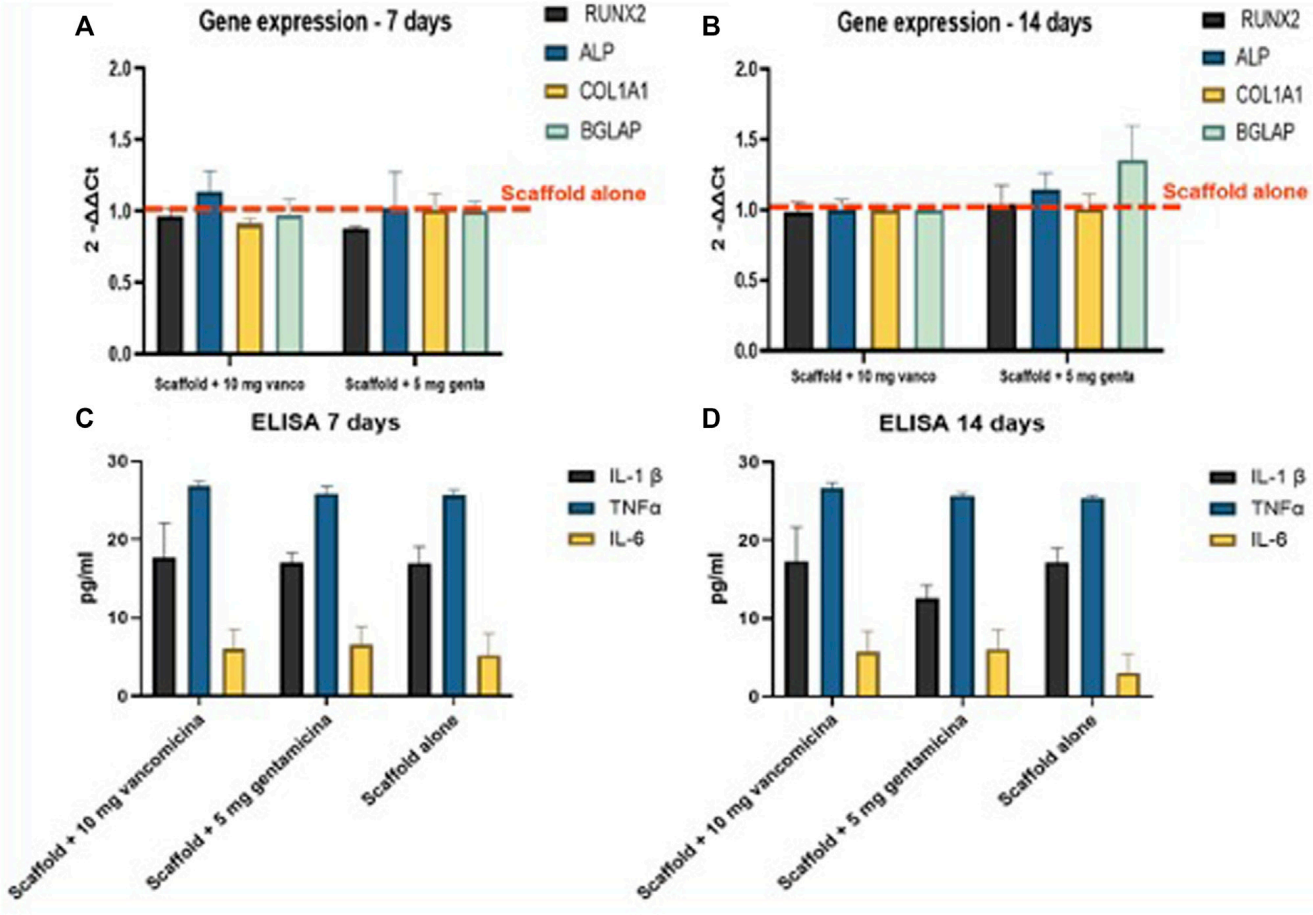
**(A,B)** Realtime PCR results of the major osteoblastic lineage genes conducted on hMSC in the 3D models at **(A)** 7 and **(B)** 14 days; RNA was extracted and the relative gene expression of RUNX2, ALPL, COL1A1 and BGLAP was calculated by normalizing to a housekeeping gene (GAPDH). **(C,D)** Evaluation of pro-inflammatory growth factors, Interleukin 1 beta (IL1β), Tumor Necrosis Factor alpha (TNFα) and Interleukin 6 (IL6) secreted by the 3D fracture models in the culture medium and assayed by immunoenzymatically tests after **(C)** 7, and **(D)**14 days of culture.

All 3D models were evaluated at 7 and 14 days of culture investigating the expression of the most common genes related to osteoblast activity and differentiation. In detail, RUNX2, ALPL, COL1A1, BGLAP gene expression in the different 3D models have not shown significant differences in comparison to the unloaded scaffolds (Figures 6A, B).

Levels of secreted pro-inflammatory cytokines, (Figures 6C, D), IL1β, TNFα and IL6 have not shown significant difference between the scaffolds loaded with 10 mg of vancomycin hydrochloride or 5 mg of gentamicin sulfate or unloaded.

### 3.4 Antibacterial activity

**FIGURE 7:**
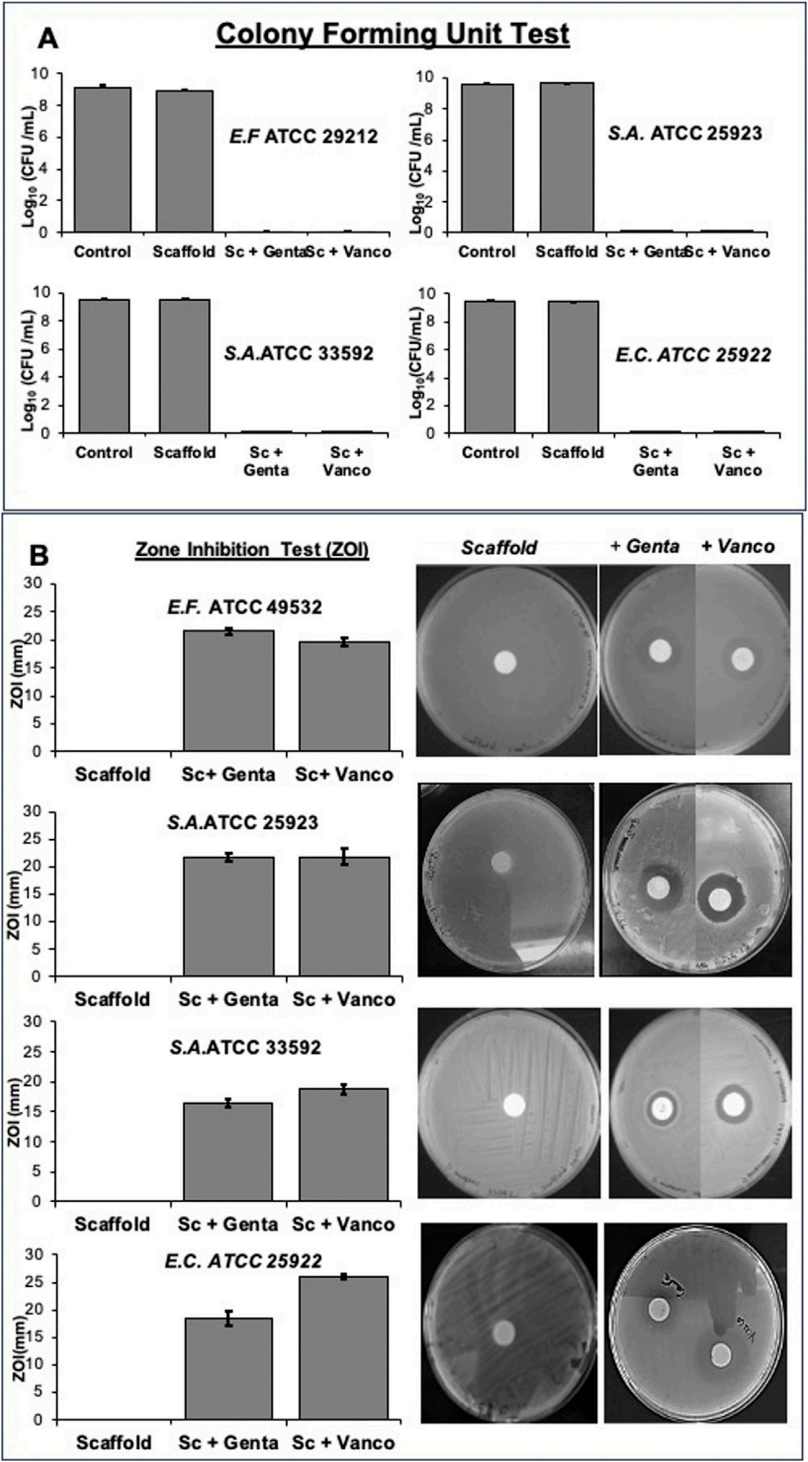
Growth bacterial inhibition was determined using both Gram-positive bacterial strains (*Staphylococcus aureus* (ATCC 25923), *Staphylococcus aureus* (ATCC 33592), *Enterococcus faecalis* (ATCC 29212) and the Gram-negative bacterial strain *Escherichia coli* (ATCC 25922) by Colony forming unit test **(A)** and Zone inhibition test **(B)** on scaffold loaded with 5 mg of gentamicin sulphate or 10 mg of vancomycin hydrochloride or unloaded disc.

The antimicrobial analysis demonstrated as scaffold loaded with gentamicin and vancomycin exerted antibacterial properties, inhibiting bacterial growth and avoiding biofilm formation through the release of the various loaded antibiotics. In particular, the growth bacterial inhibition (Figure 7) was evidenced by the colony forming unit test (Figure 7A) and the zone inhibition (ZOI) test (Figure 7B). Figure 7A shows that the unloaded scaffolds had no bactericidal action, as the results were superimposable on those of the control culture. In contrast, scaffolds loaded with gentamicin and vancomycin slowly released the antibiotics into the culture medium, causing the death of the various bacterial strains tested. In Figure 7B, is evident that the unloaded scaffolds had no antibacterial capacity, as no halo of growth inhibition was produced, which instead was observed where the bacteria were in contact with the scaffolds loaded with gentamicin and vancomycin.

**FIGURE 8:**
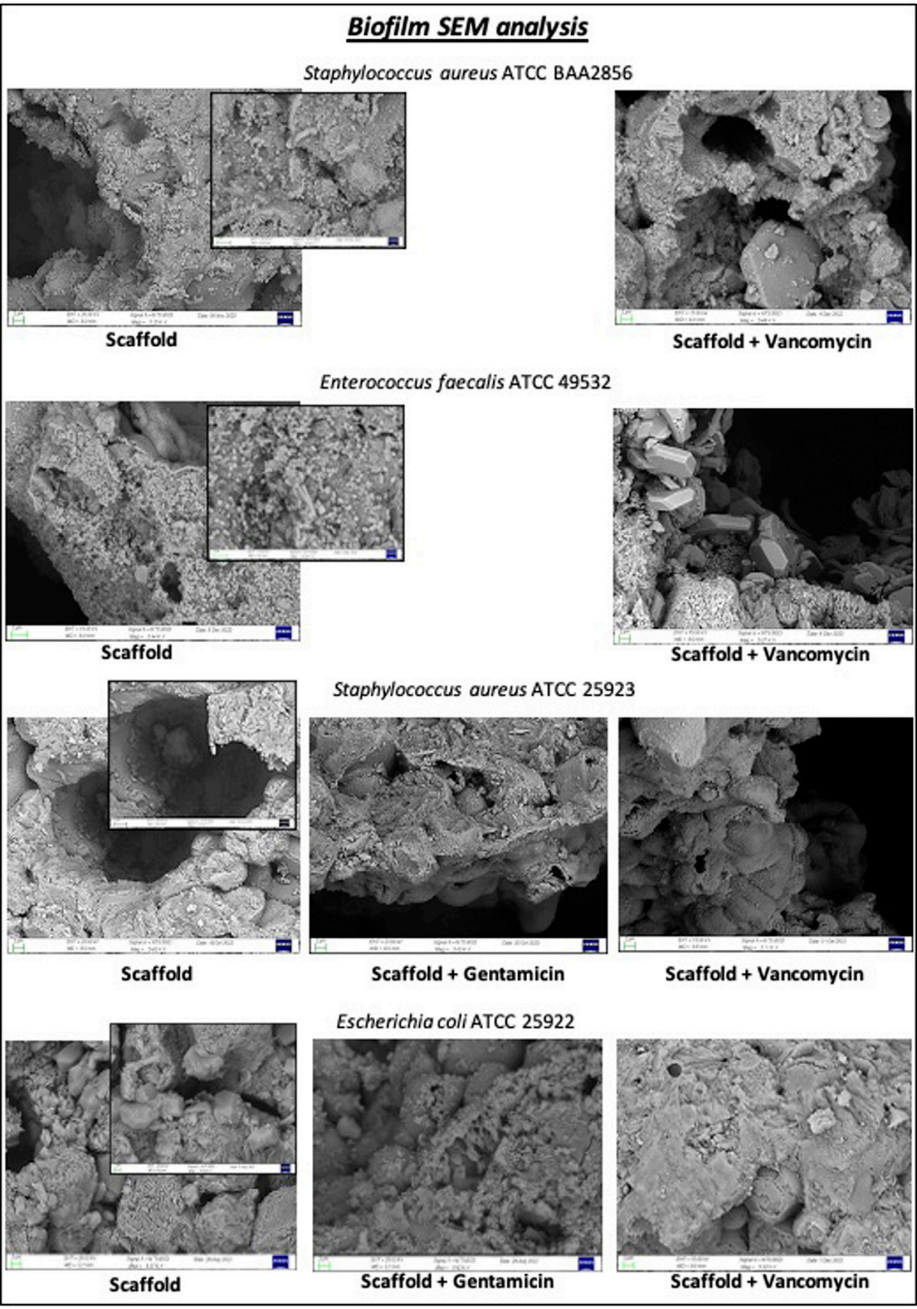
Biofilm inhibition test was performed on disc loaded with 5 mg of gentamicin sulphate or 10 mg of vancomycin hydrochloride or unloaded scaffold for each bacterial strain after 24 h. SEM images showed a reduction of biofilm when each bacterial strain was seeded on scaffold loaded with gentamicin or vancomycin for 24 h.

On the other hand, the antibiotics gentamicin sulfate or vancomycin hydrochloride loaded on the scaffold exert a strong inhibitory effect on the biofilm formation ability of different bacterial strains after 24 h, as showed in Supplementary Figure S2. The SEM micrographs of Figure 8 have confirmed the inhibitory effect of scaffold loaded. Strong bacterial colonization with matrix formation is observed in the unloaded scaffold, while the presence of bacteria is significantly reduced in scaffolds loaded with gentamicin or vancomycin.

## 4 Discussion

Bone defects can result from malformations, high-energy trauma, bone resection for various pathologies such as tumors or infections, or the treatment of complex non-unions, and are a major challenge in orthopaedic practice (Roffi et al., 2017; Xue et al., 2022). More than 10% of all reconstructive skeletal procedures involve bone grafting. It is estimated that two to three million grafts are performed worldwide each year (Campana et al., 2014; Norris et al., 2021). Autograft, allograft, synthetic bone graft substitutes and bioactive glasses are the treatments used today, but other effective solutions are currently being identified to treat serious diseases that result in severe bone loss (Nauth et al., 2018; Kim et al., 2020).

Biomorphic calcium phosphate bone scaffold (b.Bone™, GreenBone Ortho S. p.A.) is a biomaterial that has shown great biocompatibility, resorption and absence of cytotoxicity in *in vitro* tests (Tampieri et al., 2018; Ruffini et al., 2021; Tavoni et al., 2021) good integration with the host tissue favoring a clear repair process in the animal model (Filardo et al., 2020; Kon et al., 2021). Recently, the clinical performance of the b. Bone scaffold was demonstrated in two separate studies: one focusing on iliac crest reconstruction after tricortical bone graft harvesting (Alt et al., 2023), and the other on the reconstruction of severely depressed plateau fractures (Tosounidis and Pape, 2023). Both case reports concluded that b. Bone represents a valuable option for restoring bone defects, with consistently good clinical and radiological outcomes.

Despite the good performance of the scaffold, the current clinical need requires further efforts on the part of the artificial bone graft to not only promote bone regeneration, but also to cope with the significant bone loss associated with bacterial contamination or infection after implantation (Domenicucci et al., 2023). Posttraumatic osteomyelitis refers to osteomyelitis that develops because of contaminated open fractures or surgical treatment of closed fractures. Posttraumatic osteomyelitis can occur in up to 25 percent of open fractures (Merritt, 1988; Gross et al., 2002). This clinical need, associated with increased morbidity, mortality, and treatment costs due to recurrent infection following bone graft implantation, is an urgent challenge for BTE and for orthopaedic surgeons (Kyriacou et al., 2020). The aim of this study was to use an *in vitro* 3D fracture model (Tschon et al., 2022) to evaluate how the functionalised scaffold can perform its function of inducing the bone regeneration process while protecting it from the serious consequences of infection.

Preliminary pharmacokinetic results indicate that the scaffold has a drug release profile that could ensure local delivery of high concentrations of antibiotics for both vancomycin and gentamicin whitin the first 24 h after implantation, probably aided by a good level of scaffold porosity. This delivery method has the potential to significantly reduces the growth of graft-related pathogens, and adhesion (Borcherding et al., 2019), which is preferable to prevent the development of antibiotic resistance and minimize the adverse effects associated with systemic antibiotic administration (Yusri et al., 2021).

The interposition of simple or antibiotic-loaded scaffolds within the 3D bone fracture model helped us simulate the *in vivo* condition by verifying that the continuous and constant release of the quantities of vancomycin and gentamicin antibiotics loaded on the scaffold does not alter the viability of hMSCs in contact with the loaded scaffolds (Figure 5A). Antibiotics loaded on the scaffold did not affect the cellular colonization of hMSCs of the scaffold interposed in the 3D bone fracture model. The live and dead staining image in Figures 5B–D shows how the hMSCs are evenly distributed, indicating a good degree of colonization of the pores after 14 culture days. The results of the gene expression analysis of genes involved in osteogenic differentiation confirm that the release of both antibiotics from the loaded scaffold has no impact on the osteogenic differentiation capability of hMSCs compared to the unloaded scaffold, both at 7 and 14 days (Figures 6A, B). These data agree with Hofman and colleagues (Hofmann et al., 2021), demonstrating that vancomycin and gentamicin show no effects on the viability, colonization capacity, or differentiation of hMSC cells. Similarly, no significant changes in the inflammatory cytokines analyzed were highlighted (Figures 6C, D). This data is comforting since the alteration of the level of proinflammatory cytokines can have a negative effect on osteoblastic differentiation (Bastidas-Coral et al., 2019; Kaplan et al., 2023).

The main challenge for the b. Bone-loaded scaffold was to effectively inhibit the growth of various bacterial strains through its antibacterial activity.

The satisfactory results obtained from the antimicrobial tests (Figure 7) show how the initial release of the antibiotic, resulting from the localization of the drug on the outer surface of the scaffold, was able to kill the different bacterial strains satisfactorily, as demonstrated by the formation of dosage colony units (Figure 7A). The second phase, characterised by slower drug release for an elongated period, likely contributed to inhibiting bacterial growth, as demonstrated by the zone inhibition test (Figure 7B) and *in vivo* could be to prevent bacterial re-infection (Abd El-Hamid et al., 2024).

This device shows promise for clinical use due to its ability to inhibit bacterial biofilm. Biofilms are aggregates of microorganisms embedded in a self-produced matrix of extracellular polymers (Urish and Cassat, 2020) and are responsible for the majority of staphylococcal bone infections, such as osteomyelitis and other orthopaedic infections (Kavanagh et al., 2018). As reported in the literature, the presence of bacteria embedded in biofilm is more resistant to systemic use of antibiotics because the biofilm reduces the local concentration of antibiotics on the bacterial surface. In fact, the use of devices that both promote tissue repair and deliver local antibiotics has the function of increasing the local concentration of antibiotics to overcome the resistance induced by the biofilm (Ferracini et al., 2018).

As illustrated in Figure 8, tested loaded scaffold meets these requirements by exerting an inhibitory effect on biofilm formation through the release of vancomycin and gentamicin. This effect is evident in the absence of bacterial colony formation upon sonication of the scaffold immersed in a bacterial culture for 24 h (Supplementary Figure S2), as well as in the SEM images revealing the absence of true biofilm (Figure 8).

These promising results have significant implications for the treatment of infected or potentially infected bone loss resulting from open fractures and non-unions. The drug-loaded scaffolds developed in this study have the potential to serve as bone scaffolds capable of delivering antibiotics to eliminate and prevent bacterial infections over an extended period. This could significantly impact clinical practice by reducing treatment times, the need for multiple surgeries, and prolonged hospital stays.

In conclusion, these results demonstrate the potential of b. Bone scaffolds to effectively address critical challenges in orthopedic surgery and patient care. The device has been shown to inhibit bacterial growth and biofilm formation through a high potency of antibiotic release in a shorter time, reducing the likelihood of therapeutic failure due to increased resistance. These scaffolds offer a promising solution for combating bone infections and improving patient outcomes. Further studies can be conducted on combining different antibiotics on these devices to enable tailored and more effective therapy against numerous pathogens that cause post-traumatic osteomyelitis.

## Data Availability

The original contributions presented in the study are included in the article/Supplementary Material, further inquiries can be directed to the corresponding author.

## References

[B1] Abd El-Hamid H. K. Farag M. M. Abdelraof M. Elwan R. L. (2024). Regulation of the antibiotic elution profile from tricalcium phosphate bone cement by addition of bioactive glass. Sci. Rep. 14 (1), 2804. 10.1038/s41598-024-53319-2 38307930 PMC10837204

[B2] Alt V. Walter N. Rupp M. Begué T. Plecko M. (2023). Bone defect filling with a novel rattan-wood based not-sintered hydroxyapatite and beta-tricalcium phosphate material (b.Bone™) after tricortical bone graft harvesting - a consecutive clinical case series of 9 patients. Trauma Case Rep. 44, 100805. 10.1016/j.tcr.2023.100805 36851907 PMC9958041

[B3] Bastidas-Coral a P. Hogervorst J. M. A. Forouzanfar T. Kleverlaan C. J. Koolwijk P. Klein-Nulend J. (2019). IL-6 counteracts the inhibitory effect of IL-4 on osteogenic differentiation of human adipose stem cells. J. Cell. Physiol. 234 (11), 20520–20532. 10.1002/jcp.28652 31016754 PMC6767193

[B4] Borcherding K. Marx D. Gätjen L. Bormann N. Wildemann B. Specht U. (2019). Burst release of antibiotics combined with long-term release of silver targeting implant-associated infections: design, characterization and *in vitro* evaluation of novel implant hybrid surface. Mater. (Basel) 12 (23), 3838. 10.3390/ma12233838 PMC6926566 31766488

[B5] Campana V. Milano G. Pagano E. Barba M. Cicione C. Salonna G. (2014). Bone substitutes in orthopaedic surgery: from basic science to clinical practice. J. Mater Sci. Mater Med. 25 (10), 2445–2461. 10.1007/s10856-014-5240-2 24865980 PMC4169585

[B6] Dastgheyb S. S. Hammoud S. Ketonis C. Liu a Y. Fitzgerald K. Parvizi J. (2015). Staphylococcal persistence due to biofilm formation in synovial fluid containing prophylactic cefazolin. Antimicrob. Agents Chemother. 59 (4), 2122–2128. 10.1128/aac.04579-14 25624333 PMC4356782

[B7] Domenicucci M. Galante C. Cavina Pratesi F. Monica M. a. T. Aloj D. C. Milano G. (2023). New bone formation using antibiotic-loaded calcium sulfate beads in bone transports for the treatment of long-bone osteomyelitis. Eur. J. Orthop. Surg. Traumatol. 33 (6), 2489–2496. 10.1007/s00590-022-03461-2 36547706

[B8] Ferracini R. Martínez Herreros I. Russo A. Casalini T. Rossi F. Perale G. (2018). Scaffolds as structural tools for bone-targeted drug delivery. Pharmaceutics 10 (3), 122. 10.3390/pharmaceutics10030122 30096765 PMC6161191

[B9] Filardo G. Kon E. Tampieri A. Cabezas-Rodríguez R. Di Martino A. Fini M. (2014). New bio-ceramization processes applied to vegetable hierarchical structures for bone regeneration: an experimental model in sheep. Tissue Eng. Part A 20 (3-4), 763–773. 10.1089/ten.TEA.2013.0108 24099033 PMC3927717

[B10] Filardo G. Roffi A. Fey T. Fini M. Giavaresi G. Marcacci M. (2020). Vegetable hierarchical structures as template forbone regeneration: new bio-ceramization processfor the development of a bone scaffold applied toan experimental sheep model. J. Biomed. Mater. Res. Part B Appl. Biomaterials 108 (3), 600–611. 10.1002/jbm.b.34414 31095882

[B11] Gross T. Kaim a H. Regazzoni P. Widmer a F. (2002). Current concepts in posttraumatic osteomyelitis: a diagnostic challenge with new imaging options. J. Trauma 52 (6), 1210–1219. 10.1097/00005373-200206000-00032 12045656

[B12] Habraken W. Habibovic P. E. Bohner M. (2016). Calcium phosphates in biomedical applications: materials for the future? Mater. Today 19 (2), 69–87. 10.1016/j.mattod.2015.10.008

[B13] Hofmann J. Klingele S. Haberkorn U. Schmidmaier G. Grossner T. (2021). Impact of high-dose anti-infective agents on the osteogenic response of mesenchymal stem cells. Antibiot. (Basel) 10 (10), 1257. 10.3390/antibiotics10101257 PMC8532700 34680837

[B14] Kaplan S. S. Ovecoglu H. S. Akkoc T. Genc D. (2023). Effect of inflammatory mediators on the differentiation potential of dental pulp stem cells *in vitro* .

[B15] Kavanagh N. Ryan E. J. Widaa A. Sexton G. Fennell J. O'rourke S. (2018). Staphylococcal osteomyelitis: disease progression, treatment challenges, and future directions. Clin. Microbiol. Rev. 31 (2), e00084. 10.1128/cmr.00084-17 PMC5967688 29444953

[B16] Kim T. See C. W. Li X. Zhu D. (2020). Orthopedic implants and devices for bone fractures and defects: past, present and perspective.

[B17] Kon E. Salamanna F. Filardo G. Di Matteo B. Shabshin N. Shani J. (2021). Bone regeneration in load-bearing segmental defects, guided by biomorphic, hierarchically structured apatitic scaffold. Front. Bioeng. Biotechnol. 9, 734486. 10.3389/fbioe.2021.734486 34646817 PMC8503888

[B18] Kyriacou H. Kamaraj A. Khan W. S. (2020). Developments in antibiotic-eluting scaffolds for the treatment of osteomyelitis treatment of osteomyelitis. Appl. Sci., 10, 2244, 10.3390/app10072244

[B19] Matar H. E. Bloch B. V. Snape S. E. James P. J. (2021). Outcomes of single- and two-stage revision total knee arthroplasty for chronic periprosthetic joint infection: long-term outcomes of changing clinical practice in a specialist centre. Bone Jt. J. 103-B (8), 1373–1379. 10.1302/0301-620x.103b8.bjj-2021-0224.r1 34334036

[B20] Mckee M. D. Li-Bland E. A. Wild L. M. Schemitsch E. H. (2010). A prospective, randomized clinical trial comparing an antibiotic-impregnated bioabsorbable bone substitute with standard antibiotic-impregnated cement beads in the treatment of chronic osteomyelitis and infected nonunion. J. Orthop. Trauma 24 (8), 483–490. 10.1097/bot.0b013e3181df91d9 20657257

[B21] Merritt K. (1988). Factors increasing the risk of infection in patients with open fractures. J. Trauma 28 (6), 823–827. 10.1097/00005373-198806000-00018 3385826

[B22] Minardi S. Corradetti B. Taraballi F. Sandri M. Van Eps J. Cabrera F. J. (2015). Evaluation of the osteoinductive potential of a bio-inspired scaffold mimicking the osteogenic niche for bone augmentation. Biomaterials 62, 128–137. 10.1016/j.biomaterials.2015.05.011 26048479

[B23] Nauth A. Schemitsch E. Norris B. Nollin Z. Watson J. T. (2018). Critical-size bone defects: is there a consensus for diagnosis and treatment? J. Orthop. Trauma 32 (Suppl. 1), S7–S11. 10.1097/bot.0000000000001115 29461395

[B24] Nelson S. B. Pinkney J. A. Chen a F. Tande a J. (2023). Periprosthetic joint infection: current clinical challenges. Clin. Infect. Dis. 77 (7), e34–e45. 10.1093/cid/ciad360 37434369 PMC11004930

[B25] Norris B. L. Vanderkarr M. Sparks C. Chitnis a S. Ray B. Holy C. E. (2021). Treatments, cost and healthcare utilization of patients with segmental bone defects. Injury 52 (10), 2935–2940. 10.1016/j.injury.2021.01.016 33514450

[B26] Pietrocola G. Campoccia D. Motta C. Montanaro L. Arciola C. R. Speziale P. (2022). Colonization and infection of indwelling medical devices by *Staphylococcus aureus* with an emphasis on orthopedic implants. Int. J. Mol. Sci. 23 (11), 5958. 10.3390/ijms23115958 35682632 PMC9180976

[B27] Roffi A. Krishnakumar G. S. Gostynska N. Kon E. Candrian C. Filardo G. (2017). The role of three-dimensional scaffolds in treating long bone defects: evidence from preclinical and clinical literature-A systematic review. Biomed. Res. Int. 2017, 1–13. 10.1155/2017/8074178 PMC5567443 28852649

[B28] Ruffini A. Sandri M. Dapporto M. Campodoni E. Tampieri A. Sprio S. (2021). Nature-Inspired unconventional approaches to develop 3D bioceramic scaffolds with enhanced regenerative ability. Biomedicines 9 (8), 916. 10.3390/biomedicines9080916 34440120 PMC8389705

[B29] Ruffini A. Sprio S. Tampieri A. (2013). Study of the hydrothermal transformation of wood-derived calcium carbonate into 3D hierarchically organized hydroxyapatite. Chem. Eng. J. 217, 150–158. 10.1016/j.cej.2012.11.107

[B30] Sabater-Martos M. Verdejo M. A. Morata L. Muñoz-Mahamud E. Guerra-Farfan E. Martinez-Pastor J. C. (2023). Antimicrobials in polymethylmethacrylate: from prevention to prosthetic joint infection treatment: basic principles and risk of resistance. Arthroplasty 5 (1), 12. 10.1186/s42836-023-00166-7 36864538 PMC9983184

[B31] Schulze A. Mitterer F. Pombo J. P. Schild S. (2021). Biofilms by bacterial human pathogens: clinical relevance - development, composition and regulation - therapeutical strategies. Microb. Cell. 8 (2), 28–56. 10.15698/mic2021.02.741 33553418 PMC7841849

[B32] Sprio S. Ruffini A. Tampieri A. (2021). Biomorphic transformations: a leap forward in getting nanostructured 3-D bioceramics. Front. Chem. 9, 728907. 10.3389/fchem.2021.728907 34557475 PMC8452985

[B33] Steadman W. Chapman P. R. Schuetz M. Schmutz B. Trampuz A. Tetsworth K. (2023). Local antibiotic delivery options in prosthetic joint infection. Antibiot. (Basel) 12 (4), 752. 10.3390/antibiotics12040752 PMC10134995 37107114

[B34] Tampieri A. Ruffini A. Ballardini A. Montesi M. Panseri S. Salamanna F. (2018). Heterogeneous chemistry in the 3-D state: an original approach to generate bioactive, mechanically-competent bone scaffolds. Biomater. Sci. 7 (1), 307–321. 10.1039/c8bm01145a 30468436

[B35] Tampieri A. Sprio S. Rufini A. Lesci I. G. Roveri N. (2009). From wood to bone: multi-step process to convert wood hierarchical structures into biomimetic hydroxyapatite scaffolds for bone tissue engineering. J. Mater. Chem. 19 (28), 4973–4980. 10.1039/b900333a

[B36] Tavoni M. Dapporto M. Tampieri A. Sprio S. (2021). Bioactive calcium phosphate-based composites forBone regeneration. J. Compos. Sci. 5 (9), 227. 10.3390/jcs5090227

[B37] Tosounidis T. H. Pape H. C. (2023). The use of a new grafting material (b.Bone™) for the management of severely depressed tibial plateau fractures: preliminary report of three cases. Trauma Case Rep. 47, 100893. 10.1016/j.tcr.2023.100893 37601554 PMC10436172

[B38] Tschon M. Boanini E. Sartori M. Salamanna F. Panzavolta S. Bigi A. (2022). Antiosteoporotic nanohydroxyapatite zoledronate scaffold seeded with bone marrow mesenchymal stromal cells for bone regeneration: a 3D *in vitro* model. Int. J. Mol. Sci. 23 (11), 5988. 10.3390/ijms23115988 35682677 PMC9180852

[B39] Urish K. L. Cassat J. E. (2020). *Staphylococcus aureus* osteomyelitis: bone, bugs, and surgery. Infect. Immun. 88 (7), e00932. 10.1128/iai.00932-19 32094258 PMC7309607

[B40] Wang W. Yeung K. W. K. (2017). Bone grafts and biomaterials substitutes for bone defect repair: a review. Bioact. Mater 2 (4), 224–247. 10.1016/j.bioactmat.2017.05.007 29744432 PMC5935655

[B41] Xue N. Ding X. Huang R. Jiang R. Huang H. Pan X. (2022). Bone tissue engineering in the treatment of bone defects. Pharm. (Basel) 15 (7), 879. 10.3390/ph15070879 PMC9324138 35890177

[B42] Yusri S. Elfana A. Elbattawy W. Fawzy El-Sayed K. M. (2021). Effect of locally delivered adjunctive antibiotics during surgical periodontal therapy: a systematic review and meta-analysis. Clin. Oral Investig. 25 (9), 5127–5138. 10.1007/s00784-021-04056-7 PMC8370941 34283285

[B43] Zelmer a R. Nelson R. Richter K. Atkins G. J. (2022). Can intracellular *Staphylococcus aureus* in osteomyelitis be treated using current antibiotics? A systematic review and narrative synthesis. Bone Res. 10 (1), 53. 10.1038/s41413-022-00227-8 35961964 PMC9374758

