# Supplementary material for: Preliminary osteogenic and antibacterial investigations of wood derived antibiotic-loaded bone substitute for the treatment of infected bone defects
Source: Front Bioeng Biotechnol. 2024 Jul 9;12:1412584. doi: 10.3389/fbioe.2024.1412584 (PMC11270025; doi:10.3389/fbioe.2024.1412584)
Supplement: Supplementary file 1 [file DataSheet1.docx]

#
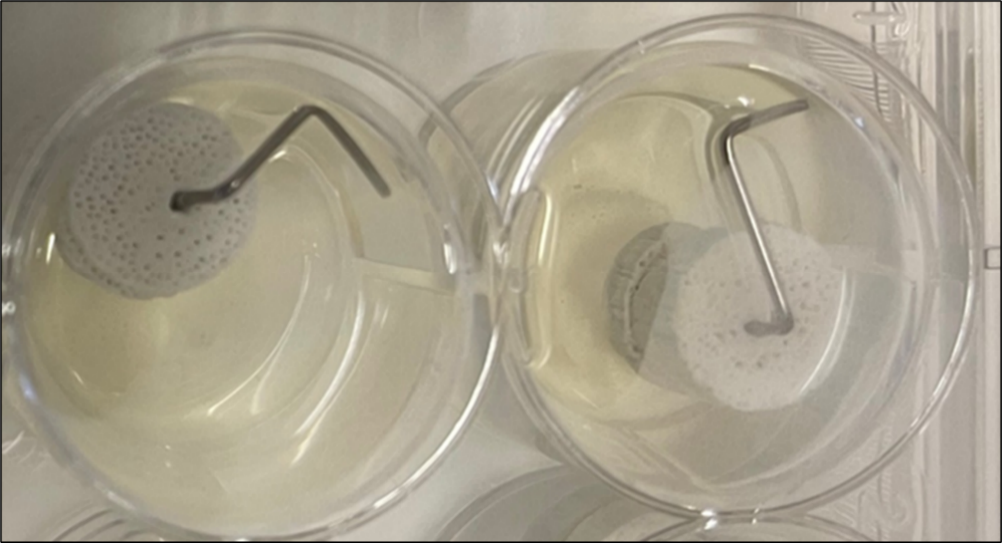
Supplementary Figure 1: Representative image of the 3D bone fracture model.

**Supplementary Table 1:** Details of primers used for gene expression analysis (QuantiTect Primer Assay, Qiagen).

| **Gene** | **Gene specification** | **Amplicon Lenght** | **Annealing Temperature** |
| --- | --- | --- | --- |
| ***GAPDH*** | Hs_GAPDH_1_SG | 95 bp | 55°C |
| ***RUNX2*** | Hs_RUNX2_1_SG | 101 bp | 55°C |
| ***ALPL*** | Hs_ALPL_1_SG | 110 bp | 55°C |
| ***COL1A1*** | Hs_COL1A1_1_SG | 118 bp | 55°C |
| ***BGLAP*** | Hs_BGLAP_1_SG | 90 bp | 55°C |

**Supplementary Table 2**. XRD results for selected peaks with Miller indices (hkl), 2 θ values.

| **No.** | **Pos. [°2θ]** | **Attibution** | **Miller Indices (hkl)** |
| --- | --- | --- | --- |
| 1 | 10,894 | HA | 010 |
| **2** | **13,792** | **β-TCP** | **104** |
| 3 | 16,905 | HA | 011 |
| **4** | **17,182** | **β-TCP** | **110** |
| 5 | 18,900 | HA | 110 |
| 6 | 21,819 | HA | 020 |
| 7 | 22,915 | HA | 111 |
| 8 | 25,915 | HA | 002 |
| 9 | 28,148 | HA | 012 |
| 10 | 28,946 | HA | 120 |
| **11** | **29,960** | **β-TCP** | **300** |
| **12** | **31,300** | **β-TCP** | **20-10** |
| 13 | 31,806 | HA | 121 |
| 14 | 32,231 | HA | 112 |
| 15 | 32,924 | HA | 030 |
| 16 | 34,107 | HA | 022 |
| **17** | **34,672** | **β-TCP** | **220** |
| 18 | 35,567 | HA | 031 |
| **19** | **35,946** | **β-TCP** | **2110** |
| 20 | 39,229 | HA | 122 |
| 21 | 39,829 | HA | 310 |
| 22 | 41,992 | HA | 311 |
| 23 | 43,899 | HA | 113 |
| 24 | 45,430 | HA | 023 |
| 25 | 46,698 | HA | 222 |
| **26** | **47,379** | **β-TCP** | **4010** |
| 27 | 48,115 | HA | 312 |
| 28 | 48,625 | HA | 320 |
| 29 | 49,494 | HA | 123 |
| 30 | 50,487 | HA | 231 |
| 31 | 51,282 | HA | 410 |
| 32 | 52,105 | HA | 042 |
| 33 | 53,217 | HA | 004 |
| 34 | 55,930 | HA | 232 |
| 35 | 57,158 | HA | 133 |
| 36 | 58,038 | HA | 051 |
| 37 | 59,953 | HA | 420 |
| 38 | 60,477 | HA | 331 |
| 39 | 61,690 | HA | 214 |
| 40 | 63,036 | HA | 331 |
| 41 | 64,012 | HA | 034 |
| 42 | 65,026 | HA | 151 |
| 43 | 66,410 | HA | 413 |
| 44 | 71,608 | HA | 341 |
| 45 | 72,238 | HA | 250 |
| 46 | 74,037 | HA | 423 |
| 47 | 74,970 | HA | 324 |
| 48 | 75,587 | HA | 125 |
| 49 | 77,005 | HA | 414 |
| 50 | 78,162 | HA | 522 |

# Supplementary Table 3: Vancomycin and gentamicin values calculated as a percentage of the initial drug load (1000ug) at each time point (Mean ± SD, n=3).

| **Timepoint** | **Vancomycin released (%)** | **Gentamicin released (%)** |
| --- | --- | --- |
| 30 min | 14.6 ± 5.6 | 3.4 ± 0.04 |
| 1h | 22.6 ± 8.5 | 7.9 ± 2.9 |
| 2h | 35.1 ± 8.9 | 35.2 ± 6.4 |
| 4h | 49.6 ± 3.0 | 60.9 ± 3.1 |
| 6h | 50.7 ± 3.3 | 67.1 ± 3.2 |
| 24h | 67.6 ± 7.1 | 75.9 ± 3.7 |
| 48h | 71.5 ± 3.4 | 84.0 ± 1.8 |
| 168h | 103.5 ± 6.9 | 100.2 ± 2.1 |
| 336h | 103.5 ± 6.9 | 100.2 ± 2.1 |


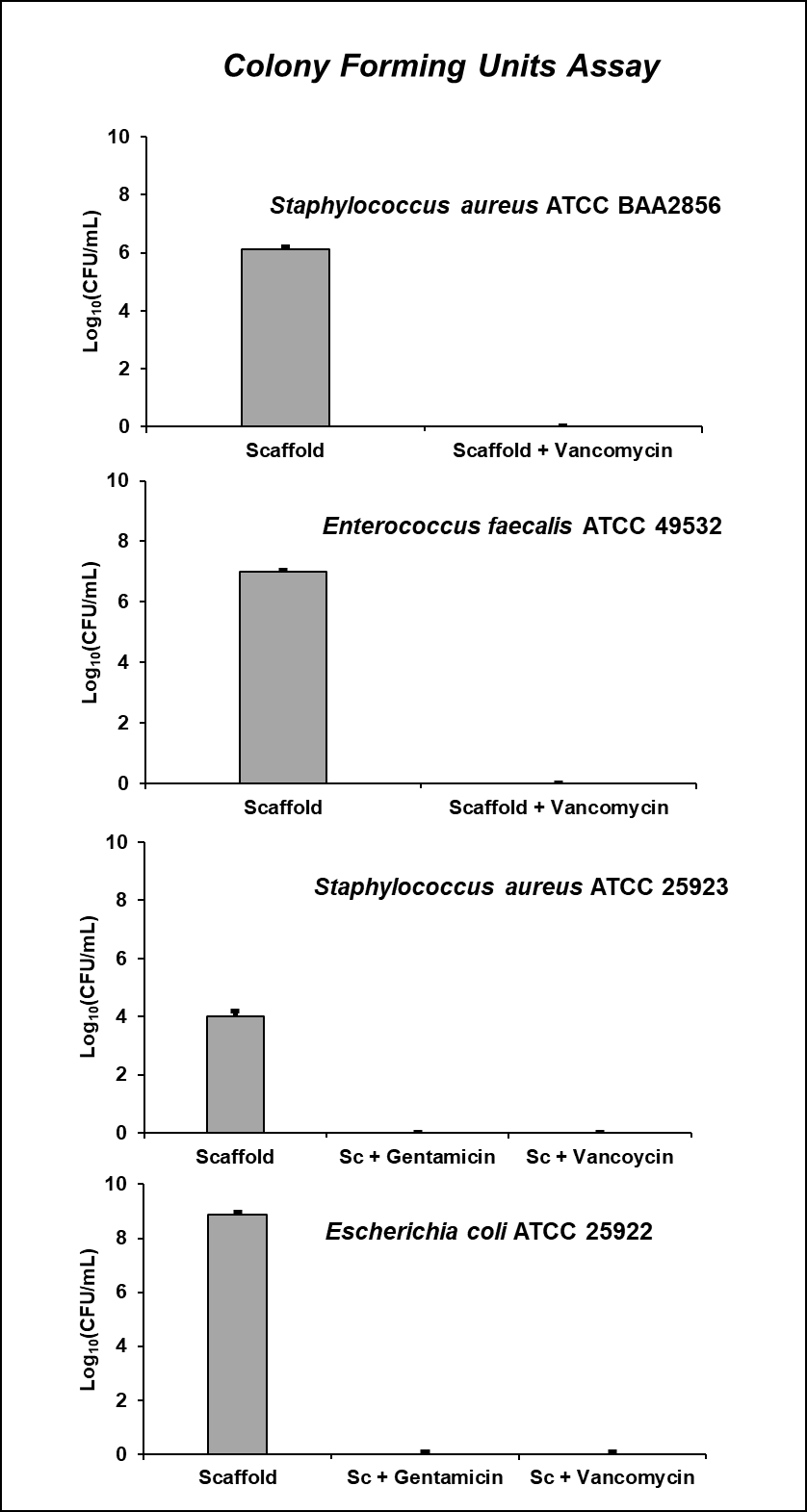


**Supplementary Figure 2.** Biofilm inhibition test was performed on disc loaded with 5 mg of gentamicin sulphate or 10 mg of vancomycin hydrochloride or unloaded GB disc for each bacterial strain after 24h. A figure, each graph summarizes the colony forming unit (CFU) count after removal of adherent bacteria from the disc at each time point.
